# Factors influencing staff attitudes to COVID-19 vaccination in care homes in England: a qualitative study

**DOI:** 10.1186/s12913-023-10031-7

**Published:** 2023-10-06

**Authors:** Bettina Friedrich, Gillian Forbes, Arnoupe Jhass, Fabiana Lorencatto, Laura Shallcross, Vivi Antonopoulou

**Affiliations:** 1 Institute of Health Informatics, Faculty of Population Sciences, University College London, 222 Euston Road, London, NW1 2DA UK; 2 Centre for Behaviour Change, Department of Clinical, Education and Health Psychology, University College London, 1-19 Torrington Place, London, WC1E 7HB UK; 3 NIHR Policy Research Unit in Behavioural Science, Population Health Sciences Institute, Newcastle University, Newcastle, NE2 4AX UK

**Keywords:** Care homes, COVID-19 vaccine, COVID-19 pandemic, Care home employees, Mandatory vaccination, Behavioural influences

## Abstract

**Background:**

The COVID-19 pandemic disproportionately affected people living and working in UK care homes causing high mortality rates. Vaccinating staff members and residents is considered the most effective intervention to reduce infection and its transmission rates. However, uptake of the first dose of the COVID-19 vaccine in care homes was variable. We sought to investigate factors influencing uptake of COVID-19 vaccination in care home staff to inform strategies to increase vaccination uptake and inform future preparedness.

**Methods:**

Twenty care home staff including managerial and administrative staff, nurses, healthcare practitioners and support staff from nine care homes across England participated in semi-structured telephone interviews (March-June 2021) exploring attitudes towards the COVID-19 vaccine and factors influencing uptake. We used thematic analysis to generate themes which were subsequently deductively mapped to the Capability, Opportunity, Motivation-Behaviour (COM-B) model. The Behavioural Change Wheel (BCW) was used to identify potential intervention strategies to address identified influences.

**Results:**

Enablers to vaccine uptake included the willingness to protect care home residents, staff and family/friends from infection and the belief that vaccination provided a way back to normality (reflective motivation); convenience of vaccination and access to accurate information (physical opportunity); and a supporting social environment around them favouring vaccination (social opportunity). Barriers included fears about side-effects (automatic motivation); a lack of trust due to the quick release of the vaccine (reflective motivation); and feeling pressurised to accept vaccination if mandatory (automatic motivation).

**Conclusions:**

We identified influences on COVID-19 vaccine uptake by care home staff that can inform the implementation of future vaccination programmes. Strategies likely to support uptake include information campaigns and facilitating communication between staff and managers to openly discuss concerns regarding possible vaccination side effects. Freedom of choice played an important role in the decision to be vaccinated suggesting that the decision to mandate vaccination may have unintended behavioural consequences.

**Supplementary Information:**

The online version contains supplementary material available at 10.1186/s12913-023-10031-7.

## Background

Care homes in England have been disproportionately affected by the COVID-19 pandemic with high rates of infection and excess mortality in residents [1, 2] and staff members [3, 4]. In May 2020, care home residents accounted for nearly 54% of all COVID-19 related deaths [5]. While there was substantial evidence in terms of epidemiological data of the adverse impact of COVID-19 on care homes residents [1, 6–8], there was much less evidence into the impact of the pandemic on employees, their working practices and their attitudes towards the prospective vaccination [9, 10].

With increasing knowledge of how infections spread and the role of asymptomatic transmission [11], ways of working in care homes changed in response to the pandemic [12]. For example, since June 2020, care home residents and staff have been offered regular testing for SARS-CoV-2 using nasopharyngeal swabs, which has made it easier for care homes to detect, control and reduce the spread of COVID-19 infection [13]. A range of public health disease control measures have been introduced such as visitor restrictions [9, 14, 15]. However, the most significant intervention to reduce infection and its transmission was the development of vaccines [16–19].

In the UK, care home staff and residents were prioritised for vaccination against COVID-19, which began with the Pfizer (BNT162b) vaccine on December 8th 2020, and was shortly followed by approval of the AstraZeneca (ChAdOx1) vaccine [20]. The majority of residents received a two-dose primary vaccine course and have subsequently received a third dose (booster vaccination). However, at the time of this study (March-June 2021), uptake of vaccination by care home workers was variable [10, 21], and this was of significant public health concern. The Department of Health and Social Care (DHSC) reported that SAGE’s social care working group advice was that 80% of staff and 90% of residents needed to have had their first dose of the vaccine to provide a minimum level of protection against outbreaks of COVID-19. However, only 53% of homes with residents over 65 in England were meeting this threshold in the first few months of the vaccine rollout. By April 2021, the staff vaccination rate was below 80% in 89 local authority areas – more than half – and all 32 London boroughs, and there were 27 local authority areas with a staff vaccination rate below 70%, based on DHSC reports [21–23].

In terms of the broader COVID-19 cases and public health measures in the UK, right before this study was conducted in 2021, the percentage of positive tests in the population was 6.5% (158 per100,000), as reported by Public Health England [24]. By mid-April 2021, the recorded number of deaths for the general population related to COVID-19 had reached over 127,000 in the UK [25].

At the time of the study, there was no lockdown and no government restrictions on non-household mixing. However, personal protective behaviours such as mask wearing, social distancing and the use of hand sanitizers were still strongly recommended. Requirements for traveling abroad included proof of vaccination status (or of vaccination exemption) in addition to a negative PCR test. A more detailed timeline of the UK lockdowns and restrictions can be found at [https://www.instituteforgovernment.org.uk/sites/default/files/timeline-lockdown-web.pdf].

Several studies in different countries have been conducted to explore concerns and population characteristics associated with vaccination uptake [26–28]. A high number of studies focused on examining vaccination intentions and behaviour in the general public to control virus transmission [29–32]. Some research has also examined healthcare workers’ vaccine attitudes and behaviours, as healthcare workers play a key role in limiting the COVID-19 transmission impact by acting as a vector for potential transmission between patients or care facilities, by role modelling for preventive behaviours, and also, helping vaccinate others [33–36]. However, very few studies examined vaccination attitudes and beliefs of social care workers working in care homes, particularly in the UK context [10, 17, 37]. Those that focused on care homes found that care home workers’ attitudes towards COVID-19 vaccination varied, with some studies reporting that the majority of social care staff had no reservations to get vaccinated immediately while others reported that staff wanted to wait or were reluctant to accept the vaccine [3, 10, 37, 38].

However, these studies did not use a theory-informed approach to study vaccine uptake. Vaccination is a form of human behaviour, which could thus be understood through application of behavioural science. The benefits of drawing on theory to investigate influences on behaviour and inform intervention development are widely recognised and emphasised in best practice intervention development and evaluation frameworks, such as the MRC Complex Interventions Guidance [39, 40]. Behavioural science theories provide explicit statements regarding processes hypothesised to regulate behaviour, which can in turn be used to explain and predict behaviours. Using theory enables drawing from, and contributing to, the wider literature and evidence-base on what influences behaviour change [41]. One simple, integrated model of behaviour change is the COM-B model, which posits that in order for a desired behaviour to occur, the individual must have the Capability (physical and psychological), Opportunity (physical and social) and Motivation (reflective and automatic) to do so [42]. The model has been used to explain potential influences on a number of protective behaviours related to COVID-19, including vaccination uptake in the general population [43–46]. The COM-B model is mapped to frameworks such as the Behaviour Change Wheel and Behaviour Change Techniques taxonomy [47, 48] to suggest which types of behaviour change strategies are more likely to be effective in addressing influences within different domains of capability, opportunity and motivation. This facilitates more systematic and transparent step-wise progression from understanding factors influencing behaviour to generating concrete suggestions for how best to change the behaviour of interest. However, to our knowledge, behavioural science frameworks have not been applied to investigate factors influencing COVID-19 vaccination uptake in social care professionals working in care home settings, and to generate suggestions for intervention approaches to support vaccine uptake in this context.

Therefore, the present study aimed to apply behavioural science frameworks to identify influences on COVID-19 vaccination uptake amongst staff working in UK care homes. Identifying such influences is a pre-requisite for addressing vaccine hesitancy and enabling future preparedness. Consequently, a secondary aim was to generate recommendations for potential behaviour change intervention strategies to support vaccine uptake.

The specific research questions addressed were:

1. Which factors related to capability, opportunity, and motivation influence care home staff’s decision to be vaccinated against COVID-19?
2. Which behaviour change strategies could help target key reported influences in order to reduce vaccine hesitancy and support uptake?

## Methods

### Study design

This study was part of the larger VIVALDI programme of research [16, 49], which collected and analysed epidemiological data on the impact the COVID-19 in care homes residents and staff in England. VIVALDI included a larger number of participants from a wide selection of care homes. This study was part of a qualitative explorative work package, which recruited staff employed in care homes that were already participating in VIVALDI.

### Participants and recruitment

Three large care home providers in England who were participating in the VIVALDI study [49] were asked to identify care homes that might be willing to take part in interviews (convenience sampling). Within participating care homes, a purposive sampling approach was used to sample individual staff to take part. We aimed for the overall sample to include representation of as wide a range of staff roles in care homes as possible. This included both staff working in clinical roles (e.g., care assistants, nurses), as well as managers and non-clinical staff (e.g., kitchen, domestic, reception staff). Participants were offered an incentive of £50 for taking part.

### Procedure

All interviews were conducted between March and June 2021 by four researchers (BF, VA, GF, AJ). Care home managers were asked to circulate a study advert and information sheet to staff. Staff who were potentially interested in participating were contacted by a study researcher to arrange an interview at a time and date that was of convenience to them. Participants provided consent to the study by signing a consent form prior to commencing the interview, and in one occasion one participant provided verbal consent at the start of the interview and completed and retuned the consent form to researchers after the interview. All interviews were conducted virtually using Microsoft Teams. Audio-recordings of interviews were transcribed verbatim and anonymised so that no individual or care home facility could be identified from the data.

### Interview guide

Data were collected using a semi-structured interview guide (see Appendix). This paper only reports the data extracted from the transcripts related to vaccine uptake. The findings from other sections of the interview guided will be reported elsewhere. The section on vaccine uptake included questions on whether or not participants had taken the vaccine (uptake), and factors potentially influencing the decision to be vaccinated or not (i.e., barriers and enablers). These questions were structured around the domains of the COM-B model, whereby the topic guide included at least one question per domain (Capability, Opportunity, Motivation) in order to ensure the broad range of potential influences on vaccine uptake were being considered. Questions were asked in an open, non-leading way, with unstructured, flexible follow-on prompts used as necessary to further unpack participants’ initial responses.

### Data analysis

A combined inductive thematic and deductive framework analysis approach was conducted [50, 51] We first conducted an inductive thematic analysis to generate themes from the transcripts regarding perceived barriers and enablers to vaccination uptake, without being restricted by the pre-defined domains of the COM-B model. Meaningful chunks of text were extracted from transcripts and descriptive summaries generated. These descriptive summaries were added to a data extraction matrix, alongside supporting and illustrative quotes. Similar in content summaries were then grouped together, and an inductive theme label was generated. Themes were classified as either a barrier to vaccination uptake, an enabler to uptake, or a mixed influence (i.e., reported as a barrier for some participants and an enabler for others). Inductively generated themes were then subsequently deductively coded to the domains of the COM-B model they were judged to best represent.

Four researchers (BF, VA, GF, AJ) independently coded all interviews. One researcher (BF) coded the majority of interviews (11/20), while the other researchers (VA, GF, JA) coded the remaining interviews (9/20). Additionally, seven out of the twenty interviews were coded by two researchers.. All researchers had training and experience in qualitative data analysis. Any disagreements regarding coding were discussed in consensus meetings with the involved coders until an agreement on the coding was reached.

Following identification of barriers and enablers, we consulted the Behaviour Change Wheel [47] to generate suggestions for potential interventions to address reported barriers and enablers and increase vaccine uptake in this context. The Behaviour Change Wheel summarises 9 broad intervention strategies for changing behaviour (i.e., education, training, persuasion, enablement, incentivisation, coercion, restriction, environmental restructuring, and modelling). These are further broken down into component behaviour change techniques (BCTs), defined as the ‘active ingredients’ of a behaviour change intervention [48, p.82]. The Behaviour Change Technique Taxonomy v1 defines 93 unique BCTs. Both the Behaviour Change Wheel and Behaviour Change Technique Taxonomy have been mapped to COM-B, in matrices which suggest which types of intervention strategies are likely to be more relevant and effective in addressing barriers and enablers within Capability, Opportunity and Motivation [52, 53]. We consulted these matrices to identify potential interventions to increase vaccine uptake based on our mapping of presently identified barriers and enablers to the domains of COM-B.

### Ethics

Ethical approval for this study was granted by the University College London (UCL) Ethics Committee (Project ID: 13,355/002).

## Results

### Participants

We interviewed twenty participants from nine care homes, representing different roles, level of experience, and managerial responsibility within the care home. Participants included: heads of care (*n* = 3); care home managers (*n* = 6); care assistants (*n* = 3) nurse (*n* = 1); administrative staff (*n* = 2) and domestic staff (*n* = 5 e.g., housekeeping and kitchen staff).

The size of the nine care homes varied from 24 and 80 beds. The care homes mainly provided residential care, some had nursing units and most had specialist provision for dementia care.

Interviewees reported different experiences of the pandemic, but most had been severely affected by COVID-19 at least once during the pandemic. Some were mainly affected in wave 1 (March–May 2020), while others were badly affected in wave 2 (October 2020-January 2021). One care home lost a third of their residents during the pandemic and other care homes reported up to 75% of residents and 50% of staff became infected with COVID-19 in wave 2.

Interviews lasted on average 46 min (range: 31–66 min). All 20 interviewees reported having been offered the vaccination and having accepted it. Interviewees did not report any serious reservations about vaccination and there was broad support for its use in care homes. However, some initial hesitation was mentioned by some participants because of how quickly these vaccines were developed and their quick authorisation approval.

From the thematic analysis, five distinct but related over-arching themes regarding factors influencing vaccination uptake were generated. These themes and corresponding sub-themes were subsequently mapped onto the COM-B domains. The full set of themes, their mapping to COM-B, classification as a barrier/enabler/mixed influence, and supporting quotes are provided in Table 1. Each theme is discussed and summarised in turn below:

**Table 1:** Factors influencing the uptake of vaccination mapped to COM-B model with supporting quotes as a barrier (B) or an enabler (E), or both (Mixed)

**Factors influencing the uptake of vaccination** Direction of influence: B = barrier; E = enabler and M = barrier and enabler)	**COM-B**	**Supporting quotes**
Theme 1: Information-seeking strategies about vaccination		
- Individuals need to source information about vaccination (E)	Psy Cap	I didn’t really [research vaccination]. I just – you know, it was like a no-brainer for me
		I did a little bit of research but, you know, like I say, just there's so much in the news
- Speak with colleagues, family or friends about being vaccinated (M)	Soc Opp	My husband told me I was stupid if I didn’t have it (E)
		[I] had a conversation with one staff member who was resistant because they are vegan (B)
- Misleading informational sources about vaccination (e.g., social media) (B)	Psy Cap	There was too much scaremongering to start with that has actually put people off
- Aware of vaccines from previous experiences of having flu vaccines (E)	Psy Cap	It's a bit like I wasn't very keen on ever getting the flu jab, but then when things were explained to me properly, it made sense and I started having my flu jab
- Information about vaccination being provided by care home employer (E)	Phy Opp	They [care home management] put a poster up. There was just a poster up. It was just a poster saying, if you want to have your vaccination, phone this number
Theme 2: Ease of access to vaccination		
- Vaccine location require travelling and not always easily access by public transport (M)	Phy Opp	Originally there were issues regarding having to travel to the location for the vaccination and some staff members did not want to do this but this got resolved when local practitioner could do it (B)
		It was easy to get the vaccination through the care home (E)
- Roll out of vaccination before having time to consider being vaccinated (B)	Ref Mot	We were told like on the Tuesday we were going to be vaccinated on the Friday. So, some of them felt like it was a bit too quick to decide
Theme 3: Social interactions and support from managers, colleagues and care home organisations		
- Recommended by care staff management to be vaccinated (E)	Soc Inf	They leave it up to – I think they obviously would like you to have it, but they’re not like putting any pressure on anyone or anything, so it is up to us if we have it
- Concerns vaccination from having pre-existing health conditions (B)	Ref Mot	Some [staff]…… were concerned because of certain health conditions that they had
- Importance of being allowed to decide for yourself by care home providers (E)	Ref Mot	Most staff members are pro vaccination
		Some staff members did not see the purpose of the vaccination (Barrier)
- More targeted information for staff who are vaccine hesitant (E)	Psy Cap	Some few members of staff were sceptical at the start but are getting the vaccination now. They received a lot of information together with the jab
Theme 4: Benefits of vaccination		
- Vaccination protects you, care home residents and close contact/family against Covid (E)	Ref Mot	I understand the responsibility that comes with that, to protect them [care home residents]
		…expect my mum to look after my son …..so I can earn a living, then I need to protect her
- Vaccination is not required because Covid 19 is a fallacy or if not had Covid 19 up to now (B)	Ref Mot	Covid is just a made-up thing and no one will persuade her otherwise. I think, because she hasn’t had the virus herself, she thinks she’s invincible
- Vaccination is the only way back to normality, i.e., pre-covid (E)	Ref Mot	I want things to, well, not go back to normal but have a way, so we have a’way of going back to normal’
- Importance placed on vaccination (E)	Ref Mot	Majority of staff accepted vaccination
- Vaccination allows travel abroad either on holiday or to travel back home (E)	Ref Mot	Secondly, I want to go back to [country], and I’m hoping that we’re going to have a vaccination passport, which says, there you go, I’ve had my vaccinations, please let me back to [country]
- Concerns about safety of vaccination because of limited testing (B)	Ref Mot	I do believe one of them [staff not vaccinated, saying], there’s not been enough testing on it
		Majority of staff accepted vaccination. Some did not want the vaccination at the start bit then changed their minds when they saw that it is safe
- Vaccine hesitancy comes concerns about possible side-effects (B)	Ref Mot	I was just worried about side effects, but yeah, I’m – I feel better now – well, I had the first dose
Theme 5: Emotional response to vaccination		
- Pre-vaccination: Worried about the negative consequences of being vaccinated (B)	Aut Mot	I was just worried about side effects
- Post vaccination: A sense of relief and feel better from being vaccinated (E)	Aut Mot	I was, yeah, I was happy I got it and yes, and I was relieved. It made me feel more comfortable, so that if I came in contact with anybody it was a, yeah definitely a bit of a relief

### Theme 1: Information-seeking strategies about vaccination

Overall, this theme was an enabler to vaccination uptake. We asked participants what kind of information sources they have used to make their decision on vaccination. The majority of participants said that they either conducted their own research or based their decision to get vaccinated on information shown in the media (e.g., radio, TV) provided by the government and local hospital communications, or through discussions with colleagues and close friends/family. A few participants mentioned social media as an informational source, but there was awareness that this was a potential source of misleading information, which had contributed to many colleagues’ decisions to refuse vaccination. Most participants felt very well informed about vaccination for COVID-19. Some discussed whether to be vaccinated with friends and/or family members and other care home colleagues, particularly when vaccination roll-out started.

Most participants expressed positive attitudes towards vaccination and some mentioned that they were also having the seasonal flu vaccine to protect the residents. In this context, previous flu vaccination acted as a familiarisation factor (and hence an enabler) for exposure to vaccine information. As a result, these individuals reported that they did not require a lot of additional information for the COVID-19 vaccine.

It was acknowledged that the care home employers supplied a lot of information to their employees, which had facilitated their decision-making. Many participants mentioned that they valued the information and support that they received through the care home employer. Some participants mentioned that a confidential helpline was also set up by their provider through which care home staff could get information on vaccinations if requested.

Overall, participants felt very well-informed and none of them expressed concern about not having had sufficient information available, as illustrated in this quote:

“I don’t think there’s a lot else that can be done really. Because the information is all there. There’s opportunity if you're not sure to check with your own GPs or to chat to others…”(Deputy care home manager).

### Theme 2: ease of access to vaccination

Having to travel to another location to get vaccinated was identified as a barrier to vaccine uptake, as this made some individuals reluctant to receive the vaccine initially. However, ease of access and convenience of vaccination when vaccination was later offered in the care home was reported as an enabler. The following quote highlights this point:

“We had a doctor’s surgery came out one day and vaccinated all of us, we were all in a line.” (Head Housekeeper)

Some staff members were also taken by surprise with how quickly they were offered the vaccine and reported that they needed more time to carefully consider and process all the available information before taking the decision to be vaccinated.

### Theme 3: Social interactions and support from managers, colleagues and care home organisations

Social influences, in terms of participants’ social network and interactions, were identified as a key enabler to vaccine uptake. When participants were asked about the attitudes of their friends and family members towards vaccination, they all indicated that they had very similar attitudes to their own, as this quote shows:

“Yeah, like I said, my husband told me I was stupid if I didn’t have it. Yes. And my oldest son, you know, he – he said he was going to have it when he gets the chance. You know, it’ll be a little while yet. So yeah, they were glad, I think.” (Senior night support worker)

Some of the main reported reasons for why people who were initially reluctant to accept the vaccine changed their mind included seeing that others who had been vaccinated remained well over time, (i.e., they had not experienced any serious side effect) and positive and reassuring conversations with colleagues and with their employers.

All participants mentioned that their care home employers encouraged their staff to accept the vaccination, but none reported that this resulted in them feeling pressured by their employers to do so.

Furthermore, all participants explicitly emphasised the importance of giving people the opportunity to make up their own minds (enabler). Participants were asked about their interactions and discussions with colleagues who decided not to be vaccinated, as indicated in the following quote:

“They’re worried about it, so – but I know I spoke to one of them and they are thinking about it now because like everyone’s been okay. I think they’re just worried, again, about side effects.” (Night support worker)

They all reported encouraging colleagues to be vaccinated and to change their minds, but also emphasised that they respected their colleagues’ views and their right to choose whether to be vaccinated or not. All participants valued the fact that staff had autonomy to make their own decision. They suggested that their vaccine hesitant colleagues should receive more information and that it was important to listen to and address their concerns. They felt that pointing out that vaccination was a ‘way back to normality’ was likely to have a positive influence on staff’s decision to be vaccinated (enabler).

### Theme 4: Beliefs about benefits of vaccination

Participants clearly stated their perceived benefits of vaccination, which motivated them to get the vaccine (Enabler). Reasons provided for accepting the vaccination included staff own protection, protection of residents and close contacts/family members, leading by example and doing what is for the “greater” good, allowing more freedom in the future and believing that it was the ‘only way back to normality.’

Some participants mentioned that being vaccinated would allow them to travel abroad, which was particularly important to those staff members who were originally from a different country, as stated in the following quote:

“Well, first of all I think it’s my duty. Secondly, I want to go back to [country], and I’m hoping that we’re going to have a vaccination passport, which says, there you go, I’ve had my vaccinations, please let me back to [country].” (Care home manager)

All participants stated they were not doing anything different following vaccination. The same protective measures were still in place post vaccination (e.g., PPE and social distancing) and continued to be followed. They also stated that they would also adhere to the same personal protective measures in their lives outside of the work environment.

In contrast, when asked about their unvaccinated colleagues’ attitudes and beliefs, participants believed that concerns about potential side-effects was the main reason for their colleagues’ hesitancy (i.e., barrier to vaccine uptake). In particular, those with pre-existing health conditions were especially concerned and apprehensive that vaccines might not have been tested appropriately and that the evidence base was still very limited.

Some participants perceived that vaccine hesitancy could be more prevalent among different ethnic and religious communities who have limited trust in public health authorities, as reported in this quote:

“The ones that I found were sceptical for their ethnicity was some of our black colleagues, you know, our colleagues from Africa. I don’t know why they weren’t keen. It’s funny, the Indian individuals that we have they were really keen to, to go to [hospital] but the Black Africans weren’t that keen. But the majority of them have had it now.” (Care home administrator)

### Theme 5: Emotional response to vaccination

Emotions represented a mixed influence on vaccine uptake. Many participants mentioned that they felt happy about getting the vaccination, as indicated in this quote:

"I was quite emotional about it. I, I felt quite, I, I actually felt, I'm so lucky, I'm so lucky that I can get this vaccination, and I felt fortunate. I felt very fortunate because I know there's people in other countries who are not being given this opportunity.” (Head of Care)

One participant even reported having cried out of relief. For some others, it was less emotional, and they just felt they had to do what they had to do. One participant mentioned feeling “better about themselves” after the vaccination. Some participants reported not having any particular emotional reaction to the vaccination.

### Mapping of identified barriers and enablers to proposed interventions

Table 2 presents the use of the Behaviour Change Wheel and Behaviour Change Technique taxonomy to select appropriate interventions based on the key components of the COM-B components as a basis for future policy recommendations.

**Table 2:** COM-B influences mapped to proposed interventions using the Behaviour Change Wheel (BCW)

Factors influencing the uptake of vaccination	COM-B	Intervention type	Behaviour Change Technique	Examples of how these could be designed and delivered
Individuals need to source information about vaccination	Psy Cap	Education	- Information about social and environmental consequences	Provide details of where to locate scientifically robust evidence about vaccination that is in a comprehensible and reader-friendly format
		Enablement		
Misleading informational sources about vaccination (e.g., social media)		Persuasion		
Aware of vaccines from previous experiences of having flu vaccines			- Information on health consequences	
More targeted information for staff who are vaccine hesitant			- Salience of consequences	e.g., Provide testimonials from other care home staff
			- Action planning	
			- Credible source	
Information about vaccination being provided by care home employer	Phy Opp	Education	- Prompts/Cues	Posters displayed within care homes that contain messaging on the safety and benefits from being vaccinated, i.e., the way back to normality
		Enablement	- Restructuring the physical environment	
Vaccine location require travelling and not always easily access by public transport		Environmental restructuring		
			- Environmental restructuring	
				Organise future vaccination booster to be done in situ
Speak with colleagues, family or friends about being vaccinated	Soc Opp	Persuasion	- Credible source	Having a senior staff member to act as a ‘champion’ of vaccines by taking on a leadership role. Champions address identified apprehension about vaccination with staff who are vaccine hesitant
		Modelling	- Social support (Practical)	
Recommended by care staff management to be vaccinated		Enablement		
			- Social comparisons	
			- Problem solving	
				Consider providing resources or suggested solutions for champions to
				‘problem solve’ locally
Concerns vaccination from having pre-existing health conditions	Ref Mot	Education	- Pros and cons	Facilitating communication between staff with similar roles and from different ethnic and religious communities from within the care home to exchange views, concerns and experience of being vaccinated
		Persuasion	- Credible source	
		Modelling	- Framing /reframing	
Importance of being allowed to decide for yourself by care home providers		Enablement	- Information about health consequences	
Roll out of vaccination before having time to consider being vaccinated				
Vaccination protects you, care home residents and close contact/family against Covid				
Vaccination is not required because Covid 19 is a fallacy or if not had Covid 19 up to now				Targeted campaigns and communications
Vaccination is the only way back to normality, i.e., pre-covid				
Importance placed on vaccination				
Vaccination allows travel abroad either on holiday or to travel back home				
Concerns about safety of vaccination because of limited testing				
Vaccine hesitancy comes concerns about possible side-effects				
Pre-vaccination: Worried about the negative consequences of being vaccinated	Aut Mot	Education	- Social support (Emotional)	
		Persuasion		
			- Salience of consequences	
Post vaccination: A sense of relief and feel better from being vaccinated				
			- Information about consequences	

We propose four intervention types (i.e., education, enablement, persuasion and modelling) and nine BCTs (e.g. action planning, social support, credible source) to target individual knowledge, motivation and emotional influences on vaccine uptake. Some interventions focus on increasing the individual knowledge about vaccination and the motivation of CH staff to get vaccinated. Other interventions focus on reducing any emotional concerns from being vaccinated. Therefore, an educational-based intervention aims to increase knowledge and to address the perceived lack of credible, reliable sources of evidence about vaccination. This educational intervention can be tailored to the care home setting, for example, provide details of where to locate scientifically robust evidence about vaccination that is in a comprehensible and reader-friendly format.

Other interventions focus on increasing ease of access to vaccination centres (enablement) and improving communication and promotion of vaccination by appointing a person in a leadership role to ‘champion’ being vaccinated (persuasion and modelling). This role is to encourage staff to be vaccinated and to reduce any concerns about side-effects (e.g., not being able to work), vaccine safety and risks from having pre-existing health conditions.

## Discussion

We explored UK care home staff attitudes and behaviours towards the COVID-19 vaccine before the COVID-19 vaccine became mandatory in this work environment. Overall, our findings are well-aligned with findings from previous studies on vaccination uptake. Key factors enabling vaccination uptake included willingness to protect themselves and vulnerable others [9], having an encouraging workplace and social network [12, 37] and the expectation of return to normality [54]. Concerns about the speed of vaccine development and approval, lack of research for potential long-term effects, and concerns about side effects and allergic reactions for specific health conditions were barriers linked to vaccine hesitancy [10, 17, 28, 38].

Our study demonstrates the benefits of mapping the identified factors influencing the decision-making processes leading to vaccination acceptance or refusal in order to propose theoretically-informed strategies that could potentially increase vaccine uptake in care homes. Specifically, our findings highlight the importance of capability, opportunity and motivational influences on vaccine uptake. For psychological capability, there were no issues with understanding the pros and cons of vaccination to make an informed decision from the available information. However, some knowledge of vaccines can be from less scientifically robust sources such as social media suggesting educating staff on where to find trustworthy information is important [9, 55]. Also, a focus on educating about pre-existing health conditions or concerns regarding fertility may improve the psychological capability of informing what is perceived as a ‘a risky decision’ such as vaccination [10].

For physical opportunity, there were no real obstacles with getting the vaccine because after an initial period, all vaccinations were taking place in the care homes. Nonetheless, this highlights the importance of ensuring that vaccination is convenient and accessible in order to maximise uptake, and future vaccination interventions need to be mindful of this. For social opportunity, seeking advice and information from family and friends and work colleagues was commonplace, and had resulted in some colleagues who were initially reluctant to accept the vaccine to change their minds. This suggests that social opportunity is an enabler that should be harnessed in future interventions, with more emphasis on enabling conversations between colleagues and their managers within the CH context to understand concerns and fears of the consequences of those who are vaccine hesitant and how best to address these apprehensions [9, 37]. Especially if a positive vaccine attitude is endorsed or modelled by a senior member of staff or someone who has a leadership role within the CH or from the wider healthcare community to promote the benefits of vaccination, this can have a significant impact [26, 36, 56].

Motivation was the most complex aspect of vaccine uptake. In terms of reflective motivation, most participants prioritised being vaccinated to protect themselves and others from COVID-19 because it was the “right thing for the greater good” and knowing that everybody getting vaccinated is the way back to ‘normality’. Therefore, when targeting the beliefs of those who are vaccine hesitant, it may be valuable for persuading that vaccines are safe and effective and the optimal way forward for managing COVID-19 long-term [26]. As part of the education-based strategies, also target automatic motivation through enabling those who are vaccine hesitant to communicate their anxieties and have conversations about how best to reduce and overcome these concerns [10, 37].

Importantly, our findings highlight the role of non-judgmental, positive peer-to-peer interactions in decision making, and the need for public health authorities to address fears and safety concerns regarding the COVID-19 vaccine as a recently developed vaccine [54].

### Strengths and limitations

Strengths of this study include providing evidence to address a research gap for an under researched population and context, including a representative sample (by interviewing staff across different care homes in diverse roles) and using a theoretical framework for analysis of the data. However, an important limitation of this study is that we only interviewed individuals who had been vaccinated, which limits our ability to draw inferences about barriers to vaccination beyond perceptions of those who had been vaccinated regarding the underlying reasons for vaccine hesitancy of those colleagues who had refused the vaccination. Although findings in our study related to barriers and enablers for the BAME communities should be treated with caution, as these are not necessarily the perspective from BAME participants directly, but rather participants reporting on their perceptions of their BAME colleagues, vaccine hesitancy among the BAME communities has been well-documented in other published evidence that aligns with our findings [6, 25, 58–60].

### Policy implications and recommendations

The behavioural analysis of the findings has guided the identification of theoretically-informed proposed interventions detailing the relevant BCTs for implementation (see Table 2). In the context of recurrent discussions regarding mandatory vaccination for health and social care employees when health crises arise [59–65], we propose theory-informed implementation strategies, which would foster vaccine confidence and encourage people to get vaccinated willingly [66, 67]. Mandatory vaccination for care home staff was introduced in England on 14^th^ June 2021, and although this increased vaccine coverage, it also led a substantial minority of staff to leave the sector which exacerbated pre-existing staff shortages [21, 37, 60]. This policy does not align with our findings as people emphasised how much they valued autonomy in their decision-making process. The policy decision to mandate vaccination against COVID-19 in care home staff has now been reversed (15^th^ March 2022; https://www.gov.uk/government/publications/vaccination-of-people-working-or-deployed-in-care-homes-operational-guidance) but given the importance of regular (booster) vaccination to maintain protection against COVID-19, there is an ongoing need for evidence-informed strategies to increase vaccine uptake. In this context, our policy recommendations for the current, and for future, vaccination uptake and pandemic preparedness would be:

- Providing staff with relevant material from a credible, evidence-based source that outlines the facts around benefits and risk of getting (or not getting) the vaccination in order to allow an informed decision
- Providing easy access to vaccination (ideally on site, with no need to commute to an external location)
- Senior care home staff/ management should engage with staff about vaccination—fears of those hesitant to get vaccinated have to be taken seriously and addressed. Modelling the behaviour is also an impactful way to change negative attitudes and beliefs and lead by example.
- Facilitating communication between staff on peer level in order to exchange views, concerns and experiences regarding vaccination.
- Staff from the BAME community in particular could address concerns from their peers who are sceptical of the vaccination and “lead by example”.

## Conclusion

Overall, our research participants were positive about getting vaccinated although they reported that some colleagues were hesitant to accept the vaccination. We identified as enablers to vaccine uptake receiving adequate information material, the opportunity to get vaccination relatively hassle-free (i.e. in the care home directly), having positive conversations with care home managers and other staff about any fears as well as the awareness that being vaccinated protects people around them and that it is a way back to normalcy. The main barriers to getting vaccinated were fears of potential side effects and mistrust with regard to the development of the vaccination—especially when these were magnified by respective misinformation in social media. Based on these findings, we suggest that health and social care providers and policy makers should consider our suggested intervention strategies and care home managers, employers, and policy makers should facilitate open, non-judgmental discussions with care home workers who are vaccine hesitant to provide the opportunity to these members of staff to discuss their reasons for not getting the vaccine, while also respectfully encouraging vaccination uptake.

### Supplementary Information

**Additional file 1. **Appendix. Topic Guide questions (including all sections of the questionnaire)

## Data Availability

Data generated during the study are subject to a data sharing mandate and available in a public repository that does not issue datasets with DOIs. The fully anonymized transcripts are available here: COVID-19: Burden and Impact in Care Homes: A Mixed Methods Study, 2020–2021—ReShare (ukdataservice.ac.uk)
