# Supplementary material for: Factors influencing staff attitudes to COVID-19 vaccination in care homes in England: a qualitative study
Source: BMC Health Serv Res. 2023 Oct 6;23:1066. doi: 10.1186/s12913-023-10031-7 (PMC10557274; doi:10.1186/s12913-023-10031-7)
Supplement: Supplementary file 1 — Additional file 1. Appendix. Topic Guide questions (including all sections of the questionnaire) [file 12913_2023_10031_MOESM1_ESM.docx]

**Supplementary file**

**Appendix**. Topic Guide questions (including all sections of the questionnaire)

| **TOPIC** | **QUESTIONS** |
| --- | --- |
| **Staff role**  **and responsibilities** | *I would just like to start by asking you some general questions about yourself and your role in the care home*. |
|  | 1. **What is your current role in this care home?** 2. **How long have you been working in this role and in this care home?** 3. **How much of your role is caring for residents with Covid 19 at the moment?** 4. **Did you work in a different care home in wave one?** 5. **What type of care home do you currently work in?** *Can you describe roughly the composition of residents? Do know roughly what proportion of residents have dementia?* |
| **Impact of Covid19 on delivery of care** | *Thank you for your responses so far. I would now like to talk through how the Covid19 outbreak has impacted on your care home and the ways of working for you and your colleagues. We would like to* discuss a range of topics related to covid and non-covid care.  Let’s start with some questions about COVID19. We are particularly keen to hear about changes between wave 1 and wave 2- for example, any issues that persist, any thing that has become easier or more difficult etc*.* |
|  | 1. **In general, how is the care home managing at the moment?** 2. **In your opinion, how much of an issue has Covid19 been in this care home in wave 2? How has it been different from wave 1?** 3. **Overall, how has Covid19 impacted on ways of working in this care home?**   *Prompts: What has changed? What are you doing now that you did not do before?* **When you suspect a resident might have Covid19, what do you do?**   1. **TESTING: Are staff and residents being tested for Covid19 at the moment in the care home?**   *Prompts:* ***If not****, are there any reasons why not being tested?* ***If yes****, who is being tested? How often? Are there any challenges regarding the testing?*  *If yes to Q14*   1. **Does the care home do the testing or is it done by someone else? How many staff are required per care home to do the testing?** 2. **What sort of tests are you using?** *Are you using lateral flow immunoassays testing in your care home (or other new form of tests)?* ***If yes*** What happens if a staff or resident tests positive? Do you send a PCR swab to confirm? 3. **How long does the process of testing take?** 4. **What happens if a staff member tests positive? Do they go home immediately? Are they replaced on the shift?** 5. **If a member of staff continues to work on the floor and tests positive later, how does the care home manage a situation like this (has this actually happened?).** 6. **What impact does staff testing positive have on ways of working?** |
| **Impact on restricting visitors** | *Thank you for your responses so far. I’d now like to move on to discuss the impact of visiting restrictions* |
|  | 1. **What is the care home’s current policy and restrictions around allowing visitors for residents?** *Prompts: If visits are taking place, what do they like (e.g. are people put in pods)?* 2. ***Are visitors being tested?*** 3. **When the restrictions for visitors started, how easy/difficult was this for the care homes to action?** *Were there any challenges?* 4. **To what extent has restricting visitors impacted on your day-to-day work load?** 5. **With regard to restricting visitors or not, how do you feel the situation should be handled?** 6. **What would be risks and benefits of allowing visitors?** 7. **Do you know how residents would feel about allowing/restricting visitors? Do you know how their families would feel about this?** |
| **Covid-19 symptoms and testing of staff** | *I would now like to talk about Covid symptoms and testing* |
|  | 1. **Have you had any symptoms which you think could have been Covid19**? *If you did, what did you do?* *(Prompts: did they isolate? talked to GP? test arranged?) How at risk do you feel?* 2. **If you don’t mind me asking/sharing, have you tested positively for COVID-19 at any time point at all? You can skip this Q if you’d prefer.** *If you have tested positive, was that on routine testing or was it because you were symptomatic?* *For everybody:* *How long did it take between the test and the results? Did you still work while you were waiting for the results? How frequently are you getting tested?* 3. **How (would/did) a positive test impact on you?** *On other members of your family or household, e.g. partner, spouse work , house mate or children's school?* |
| **Vaccination** | *I would now like to ask you a couple of questions about vaccination. There are no right or wrong answers. We understand that some people are accepting the COVID-19 vaccine and others are not. We are really interested in learning more about this and hearing perspectives of both groups’.* |
|  | 1. **Have you been offered a vaccine for COVID-19?** 2. **If so, would you mind telling me whether or not you accepted the vaccine? If not, how likely would you be to accept the vaccine if offered one?** 3. **What are the reasons behind your decision*?* Which sources of information, if any, did you use to inform your decision?** 4. **How did you feel about (not) getting the vaccine?** 5. **To what extent has employer recommended or required you to be vaccinated against COVID-19?** 6. **Has it been easy for you and other the members of staff to get a vaccine?** Have there been sufficient supplies for the care home? 7. **What do others around you think about getting the vaccine?** 8. **After/if you were vaccinated did you do anything different?**   **Prompts: To what extent do you think it is necessary to physically distance or wear a face covering once you’ve been vaccinated?**   1. **What do you think could be done to support people to make decisions about taking the vaccine and to feel more comfortable taking the vaccine?** |
| **Staff morale and**  **well-being** | *I would like to now understand how working during the Covid19 pandemic has impacted on the morale and well-being of care home staff.* |
|  | 1. **What is the morale and well being of the care home staff at the moment?** *Prompts: What are the reasons for this? Has this changed between wave one and wave two?* 2. **Do you know whether there have been any issues with staff burnout or other adverse emotional reactions (anxiety, depression)?**   **prompts:** *Is there any support in place for staff?*   1. **To what extent do you feel that experiencing the current situation might make staff members consider leaving the sector?** |
| **Support requirements and lessons learnt for**  **future outbreaks** | *Some final questions about the support received and lessons learnt during this Covid19 outbreak and management of any future outbreaks* |
|  | 1. **What would you say have been the main lessons learnt during the current outbreak?** 2. **Is there anything else that could be done to better support CHs to cope with Covid19 at present and in the future?** |
| **Closing**  **Remarks** | ***Thank you – that concludes all the questions I would like to ask.***  ***Is there anything you would like to add that has not been covered by my questions so far? Or is there anything you would like to revisit or expand upon?***  ***Thank you for taking the time to speak to me*** |
